# [^18^F]-FDG-PET/CT detects subclinical optic nerve inflammation in giant cell arteritis

**DOI:** 10.3389/fimmu.2026.1802935

**Published:** 2026-03-18

**Authors:** Michael Gernert, Marc Schmalzing, Matthias Fröhlich, Yingjun Zhi, Patrick-Pascal Strunz, Hannah Labinsky, Philipp E. Hartrampf, Andreas K. Buck, Thorsten A. Bley, Konstanze V. Guggenberger, Rudolf A. Werner, Sebastian E. Serfling

**Affiliations:** 1 Department of Medicine II, Rheumatology and Clinical Immunology, University Hospital Würzburg, Würzburg, Germany; 2 Department of Otorhinolaryngology, Head and Neck Surgery, University Hospital Würzburg, Würzburg, Germany; 3 Department of Nuclear Medicine, University Hospital Würzburg, Würzburg, Germany; 4 Department of Diagnostic and Interventional Radiology, University Hospital Würzburg, Würzburg, Germany; 5 Department of Diagnostic and Interventional Neuroradiology, University Hospital Würzburg, Würzburg, Germany; 6 Department of Nuclear Medicine, LMU University Hospital, Ludwig-Maximilians-Universität (LMU) Munich, Munich, Germany

**Keywords:** [^18^F]-FDG-PET/CT, giant cell arteritis, large vessel vasculitis, optic nerve inflammation, subclinical inflammation

## Abstract

**Objective:**

Vision loss is a feared complication in giant cell arteritis (GCA). However, imaging data characterizing intraorbital inflammation and its potential predictive value for ischemic ocular complications remain limited. Such information might be helpful in clinical phenotyping and risk stratification of patients with GCA.

**Methods:**

This is a retrospective cohort study of patients with GCA, who received at least two [^18^F]-FDG-PET/CTs. Quantitative assessment included measurement of SUV_max_, SUV_mean_, and the target-to-background ratio (TBR) of both optic nerves (ONs) in the canalicular segment, serving as a surrogate for [^18^F]-FDG uptake.

**Results:**

A total of 18 patients were included; only 1 had visual symptoms. [^18^F]-FDG uptake of the ONs was higher in active GCA than in a control cohort [i.e., patients with a primary diagnosis of bronchial carcinoma; median TBR 8.0 (interquartile range 5.2–10.0) vs. 1.8 (1.6–2.5), respectively; *p* < 0.0001]. Under immunosuppressive therapy, the [^18^F]-FDG uptake of the ONs declined [median TBR of inactive GCA was 5.5 (4.3–7.2), *p* = 0.0009 vs. active GCA]. A TBR of 7.5 distinguishes between active and inactive GCA with a sensitivity of 86.7% and a specificity of 61.1% [area under the curve (AUC) 0.743, 95% confidence interval (CI) 0.574–0.912, *p* = 0.0179].

**Conclusion:**

An unexpected high uptake of [^18^F]-FDG, corresponding to inflammation of the ONs, could be detected in patients with GCA, most of whom exhibited no visual symptoms. Under immunosuppressive therapy, [^18^F]-FDG uptake declined, indicating treatment response. These findings suggest that subclinical ON inflammation in GCA may be more prevalent than previously recognized.

## Introduction

Giant cell arteritis (GCA) is the most prevalent vasculitis in individuals older than 50 years (1). GCA is a large vessel vasculitis comprising two major clinical phenotypes: Predominant involvement of the cranial arteries (cranial GCA) and predominant involvement of the aorta and arteries of the upper extremities (large vessel GCA) (2). GCA affects arteries of different sizes ranging from the aorta to small ciliary arteries. Occlusion of these ocular arteries results in arteritic anterior ischemic optic neuropathy (AAION), which can cause blindness—one of the most feared complications of GCA (3). Warning signs for vision loss are older age and ischemic symptoms, including visual symptoms (amaurosis fugax and diplopia), scalp tenderness, and jaw claudication. Moreover, elderly patients with a milder increase in inflammatory markers [C-reactive protein (CRP) and erythrocyte sedimentation rate (ESR)] are thought to be at higher risk for vision loss. To date, there are no further predictors of AAION in GCA, and data on subclinical optic nerve (ON) inflammation and/or ophthalmic arteritis are scarce (2, 4–6).

Ciliary arteries are too small to be visualized by standard imaging modalities such as magnetic resonance imaging (MRI), ultrasound, computed tomography (CT), or positron emission tomography (PET). Instead, functional imaging of the ON and its arteries may be helpful to detect subclinical intraorbital inflammation in patients with GCA. Subclinical inflammation can be detected by enhanced uptake of glucose, which can be visualized by 2-deoxy-d-[^18^F]fluoro-D-glucose (FDG) PET/CT ([^18^F]-FDG-PET/CT). [^18^F]-FDG-PET/CT is the preferred diagnostic tool for the detection of inflammation in extracranial GCA according to the European Alliance of Associations for Rheumatology (EULAR) recommendations (7), but it also detects cranial and intraorbital [^18^F]-FDG uptake. In recent MRI studies, patients with GCA with visual symptoms, as surrogate for impending vision loss, exhibited inflammation of the ON sheath in 53% (8). Additionally, a decrease of ON diameter was described by transorbital ultrasound under GCA treatment (9). Both publications show that affections of the ON can be visualized in GCA.

The objective of our study was to analyze if inflammation of the ON can also be visualized by [^18^F]-FDG-PET/CT and might thereby support the diagnosis of GCA. It should be noted that [^18^F]-FDG-PET/CT is primarily used in the assessment of large-vessel vasculitis, typically presenting with few or no cranial symptoms.

## Patients and methods

### Study design and definitions

This was a retrospective cohort study comprising all patients with GCA, who fulfilled the ACR/EULAR classification criteria of 2022 (10) and were diagnosed in our tertiary university center and received at least two [^18^F]-FDG-PET/CTs. Active disease in GCA was defined according to the EULAR consensus definition (i.e., presence of typical signs and symptoms plus at least one of the following: current activity on imaging, ischemic complications attributed to GCA, or persistently elevated inflammatory markers after other causes have been excluded) as was remission (i.e., absence of all clinical signs and symptoms due to GCA, normalization of ESR and CRP, and no evidence of progressive vessel narrowing) (11). Approval by the local ethics committee was not mandatory according to German law as this retrospective study included only clinical routine data. All data were generated in compliance with the Declaration of Helsinki.

### [^18^F]-FDG-PET/CT

#### Patient preparation and [^18^F]-FDG administration

All patients fasted for at least 6 h before imaging; water intake was permitted. Blood glucose levels were measured immediately prior to FDG injection, and examinations were performed only if glucose levels were ≤180 mg/dL. Approximately 3 MBq/kg of [^18^F]-FDG was administered intravenously. A standardized uptake time of approximately 60 min was observed before image acquisition.

#### PET/CT acquisition and image reconstruction

Imaging was performed using a hybrid PET/CT system (Siemens Biograph mCT 64 Flow edge). Whole-body PET imaging (skull base to mid-thigh) was acquired in 3D mode with 3 min per bed position. Standard corrections—including attenuation, scatter, random events, decay, and dead time—were applied automatically.

PET images were reconstructed using iterative algorithms (OSEM) with time-of-flight and point-spread-function modeling when available. Reconstruction parameters included the following: matrix size: 256 × 256, slice thickness: 2–3 mm, and Gaussian post-filter: 4 mm FWHM. Standardized uptake values (SUV_max_ and SUV_mean_) were calculated using body-weight normalization. Cross-calibration of the PET/CT system and the dose calibrator followed institutional quality assurance procedures.

#### CT protocol

A low-dose CT scan for attenuation correction and anatomical localization was acquired with the following parameters: tube voltage: 120 kVp, automated tube current modulation, slice thickness: 3 mm, and rotation time: 0.5 s. A diagnostic contrast-enhanced CT scan was performed when clinically indicated.

#### Image analysis

All PET/CT scans were reviewed by a board-certified nuclear medicine physician with more than 10 years of experience. The right and left ON were visually identified, followed by manual or semi-automated segmentation. Quantitative parameters included SUV_max_, SUV_mean_, SUV_peak_, and total lesion glycolysis (TLG). TLG values were derived for each lesion using a segmentation with a relative threshold (41% of SUV_max_). For the inflammatory assessment, regions of interest (ROIs) were placed on the ON in the canalicular segment across multiple axial slices, and mean SUV values were averaged for the evaluated segment. The target-to-background ratio (TBR) was calculated by first obtaining the background SUV_mean_ from three ROIs placed in the superior vena cava. The SUV_mean_ of the ON uptake in the canalicular segment was then divided by the average of these three vena cava measurements, yielding the TBR for each patient.

### Statistical analysis

For continuous variables, differences between paired groups were examined with Wilcoxon signed-rank tests and differences between unpaired groups were examined with Mann–Whitney *U* tests. Normal distribution of data was mainly absent. Spearman’s tests were used to calculate correlations. Receiver operator characteristic (ROC) curves were calculated to detect cutoff values with the best sensitivity and specificity. Differences were considered significant when two tailed *p*-values were less than 0.05. Excel (Microsoft, Redmond, WA) was used to collect the data. Calculations were done with Prism (V10; GraphPad Software, Boston, MA). Figures were grouped with paint.net (V5; dotPDN LLC, Kirkland, WA).

## Results

### Patients’ characteristics

A total of 18 patients with GCA were included in this study; 14 were women. The median age was 65.6 years (range: 50.1–79.0). Of these, 17 had no visual symptoms, and only 1 patient had an AAION. Congruent with that, 16 patients had a large vessel GCA type and 2 patients had a cranial GCA type in [^18^F]-FDG-PET/CT. The median CRP value was 2.6 mg/dL (range: 0–8.3) (reference < 0.5) and the median ESR was 39.0 mm/h (range: 2–120) at the time of the first [^18^F]-FDG-PET/CT. All patients received their first [^18^F]-FDG-PET/CT at the time of their GCA diagnosis and had active disease. Five patients (27.8%) were taking immunosuppressive treatment (three methotrexate and two azathioprine) and eight patients (44.4%) were taking prednisolone with a median daily dose of 0.0 mg (range: 0–80 mg, mean daily dose: 14.0 mg). Of these, 15 were in remission, i.e., had inactive GCA, at the time of their second [^18^F]-FDG-PET/CT. Three patients had a relapse at the time of their second [^18^F]-FDG-PET/CT, which is the reason why they were excluded from the inactive GCA group. A total of 13 patients (86.7%) were taking immunosuppressive treatment (4 methotrexate and 9 tocilizumab) and 8 patients (53.3%) were taking prednisolone with a median daily dose of 2.0 mg (range: 0–15 mg, mean daily dose: 3.4 mg) at the time of the second [^18^F]-FDG-PET/CT. The median time between the first and second [^18^F]-FDG-PET/CT was 1.5 years (range: 0.3–8.8 years). The [^18^F]-FDG-PET/CTs were performed between 2011 and 2024. The characteristics of the cohort are presented in **Table 1**. In the control group [19 individuals with bronchial carcinoma (BC)], 7 (36.8%) were female and the median age was 72.0 years (range: 54–86). All individuals were treatment naïve with regard to BC.

**Table 1:** Characteristics of the study population.

Characteristics	Numbers
Observation time, median (range), months	56.5 (18–91)
Female, *n* (%)	14/18 (77.8)
Age, median (range), years	65.6 (50.1–79.0)
Visual symptoms of GCA, *n* (%)	1/18 (5.6)
Large vessel GCA type (i.e., no cranial vasculitis) in [^18^F]-FDG-PET/CT, *n* (%)	2/18 (11.1)
C-reactive protein at diagnosis, median (range), mg/dL	2.6 (0.0–8.3)
Erythrocyte sedimentation rate, median (range), mm/h	39.0 (2–120)
Time between 1st and 2nd [^18^F]-FDG-PET/CT, median (range), years	1.5 (0.3–8.8)
Immunosuppressive treatment at 1st [^18^F]-FDG-PET/CT	
Methotrexate, *n* (%)	3/18 (16.7)
Azathioprine, *n* (%)	2/18 (11.1)
Prednisolone, *n* (%)	8/18 (44.4)
Prednisolone, median daily dose (range), mg	0.0 (0–80)
Immunosuppressive treatment at 2nd [^18^F]-FDG-PET/CT	
Methotrexate, *n* (%)	4/15 (26.7)
Tocilizumab, *n* (%)	9/15 (60.0)
Prednisolone, *n* (%)	8/15 (53.3)
Prednisolone, median daily dose (range), mg	2.0 (0–15)

[^18^F]-FDG-PET/CT, 2‐deoxy‐d‐[^18^F]fluoro‐D‐glucose positron emission tomography/computed tomography; GCA, giant cell arteritis.

### [^18^F]-FDG uptake of the optic nerves in patients with GCA is higher than in patients with no GCA

SUV_max_, SUV_mean_, and TBR were assessed separately for the right and left ON. For comparison of the background uptake of the ON, patients with treatment-naïve BC were chosen and means of the right and left ON were calculated. In active GCA (i.e., in the first [^18^F]-FDG-PET/CT), the [^18^F]-FDG uptake of both ONs was significantly higher than in BC. Median SUV_max_ in active GCA was 11.5 [interquartile range (IQR) 9.7–14.2] vs. 6.5 (5.1–7.3) in BC (*p* < 0.0001), median SUV_mean_ in active GCA was 7.1 (6.1–9.2) vs. 3.9 (3.2–4.5) in BC (*p* < 0.0001), and median TBR in active GCA was 8.0 (5.2–10.0) vs. 1.8 (1.6–2.5) in BC (*p* < 0.0001).

**Figure 1:**
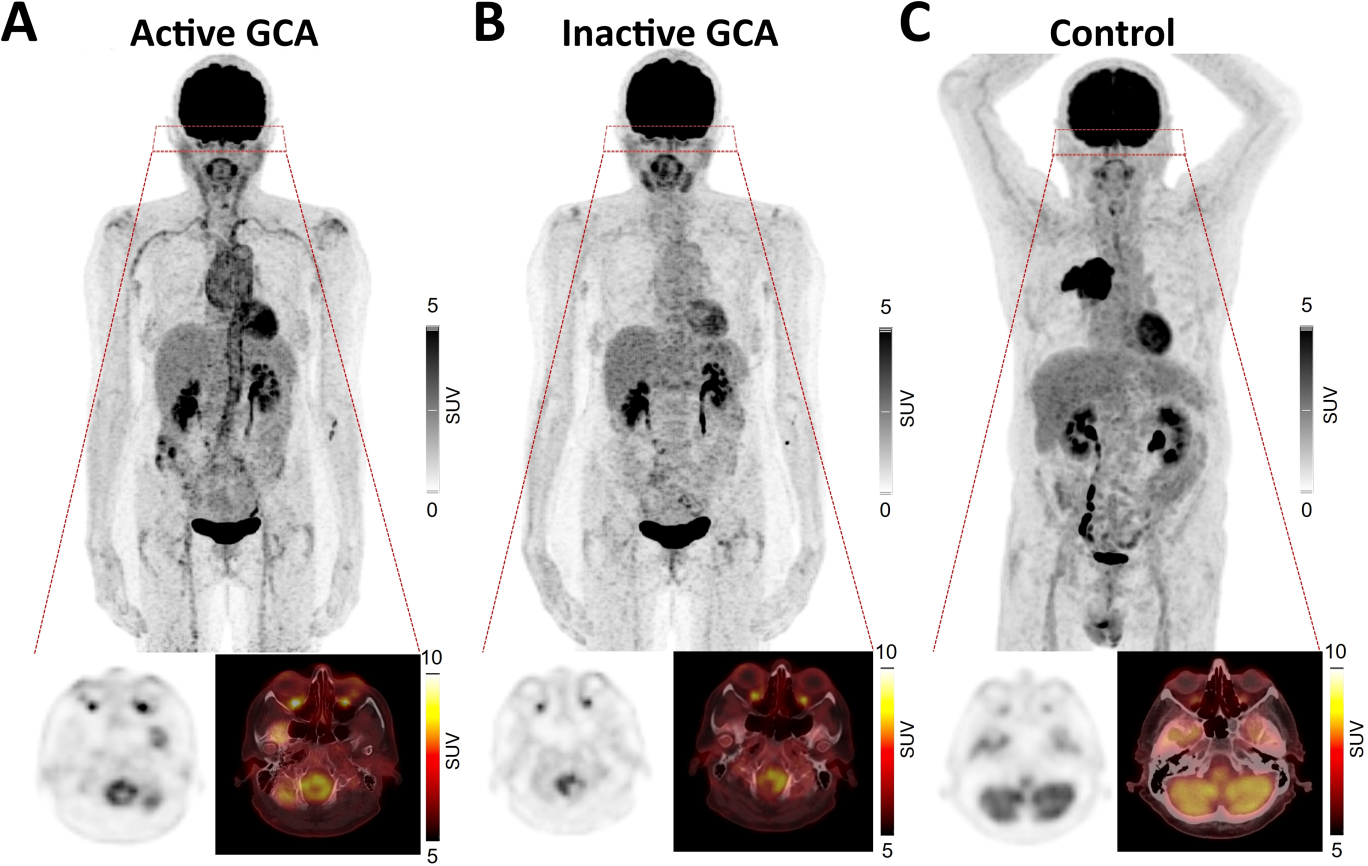
Representative increased [^18^F]-FDG uptake of both optic nerves in a patient with active GCA **(A)** without visual symptoms and hypermetabolism in projection on the aorta, subclavian, and axillary arteries. Decrease of [^18^FFDG uptake of the optic nerves in a follow-up PET/CT 16 months later under tocilizumab treatment and normalized metabolism of the aorta, subclavian, and axillary arteries **(B)**. **(C)** Representative PET/CT images of an oncologic control patient with newly diagnosed right upper lobe bronchial carcinoma and right hilar and mediastinal lymph node metastases, showing no increased [^18^F]-FDG uptake in the optic nerves, illustrating physiological background activity in the canalicular segment.

**Figure 2:**
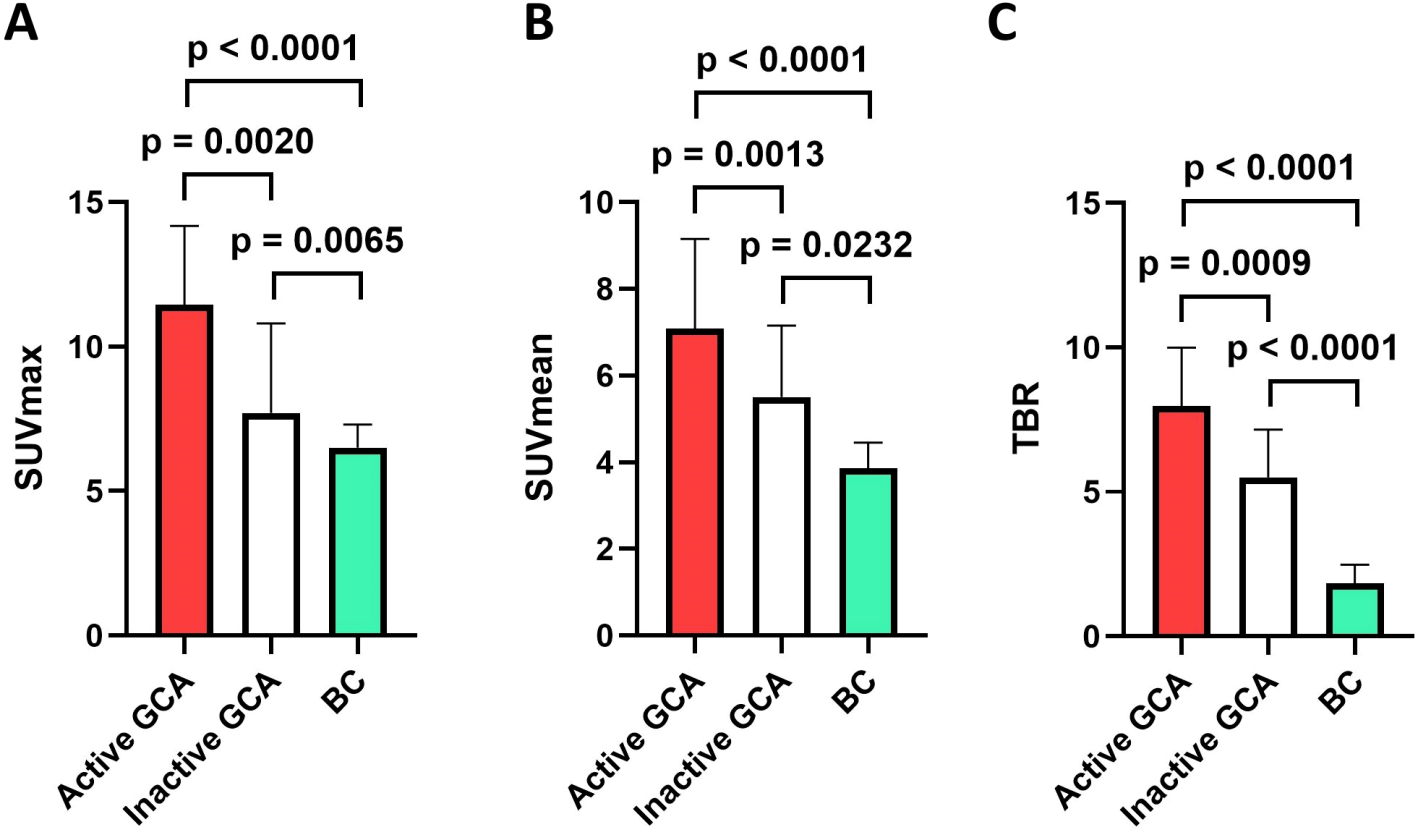
Comparison of [^18^F]-FDG uptake of both optic nerves in patients with active giant cell arteritis (GCA; red bars; *n* = 18), in inactive/treated GCA (white bars; *n* = 15), and in individuals with bronchial carcinoma (BC; green bars; *n* = 19). **(A)** shows the SUV_max_, **(B)** presents the SUV_mean_, and **(C)** shows the target-to-background ratio (TBR), which is calculated from SUV_max_/average SUV_mean_ of the vena cava. Shown are medians, and whiskers indicate interquartile ranges. *p*-values were calculated with Mann–Whitney *U* tests in active GCA vs. BC and inactive GCA vs. BC. *p*-values were calculated with Wilcoxon signed-rank tests in active vs. inactive GCA.

A representative patient with GCA with active disease and in the course of the disease with inactive GCA is shown in **Figure 1** as well as a patient from the control group with BC. A higher [^18^F]-FDG uptake compared to BC could also be detected in inactive GCA (i.e., in the second [^18^F]-FDG-PET/CT): median SUV_max_ in inactive GCA was 7.7 (7.3–10.8) vs. 6.5 (5.1–7.3) in BC (*p* = 0.0065), median SUV_mean_ in inactive GCA was 5.5 (4.3–7.2) vs. 3.9 (3.2–4.5) in BC (*p* = 0.0232), and median TBR in inactive GCA was 5.5 (4.3–7.2) vs. 1.8 (1.6–2.5) in BC (*p* < 0.0001) (**Figure 2**).

The ON [^18^F]-FDG uptake was significantly higher compared to all evaluated arteries (comprising carotids, subclavian, axillaries, truncus brachiocephalicus, ascending aorta, aortic arch, descending aorta, abdominal aorta, and iliac arteries). For example, the median SUV_max_ of the left ON was 11.4 (IQR 8.9–15.9) compared to abdominal aorta 4.2 (3.2–5.2) (*p* < 0.0001) (**Supplementary Figure 2**).

Interestingly, most of the patients had no visual symptoms. Only one patient had an AAION on the right eye with an [^18^F]-FDG uptake in the active disease state comparable to the mean of the rest of the cohort but a slightly higher uptake in the affected right eye (right ON SUV_max_ 12.0, SUV_mean_ 7.6, and TBR 9.2; left ON SUV_max_ 9.3, SUV_mean_ 5.6, and TBR 9.6).

### [^18^F]-FDG uptake of the optic nerve in patients with GCA distinguishes between active and inactive disease and decreases under immunosuppression

Patients with active (i.e., newly diagnosed) GCA had a significantly higher [^18^F]-FDG uptake of both ONs compared to inactive (i.e., sufficiently treated) GCA: median SUV_max_ in active GCA was 11.5 (9.7–14.2) vs. 7.7 (7.3–10.8) in inactive GCA (*p* = 0.0020), median SUV_mean_ in active GCA was 7.1 (6.1–9.2) vs. 5.5 (4.3–7.2) in inactive GCA (*p* = 0.0013), and median TBR in active GCA was 8.0 (5.2–10.0) vs. 5.5 (4.3–7.2) in inactive GCA (*p* = 0.0009) (**Figure 2**).

**Figure 3:**
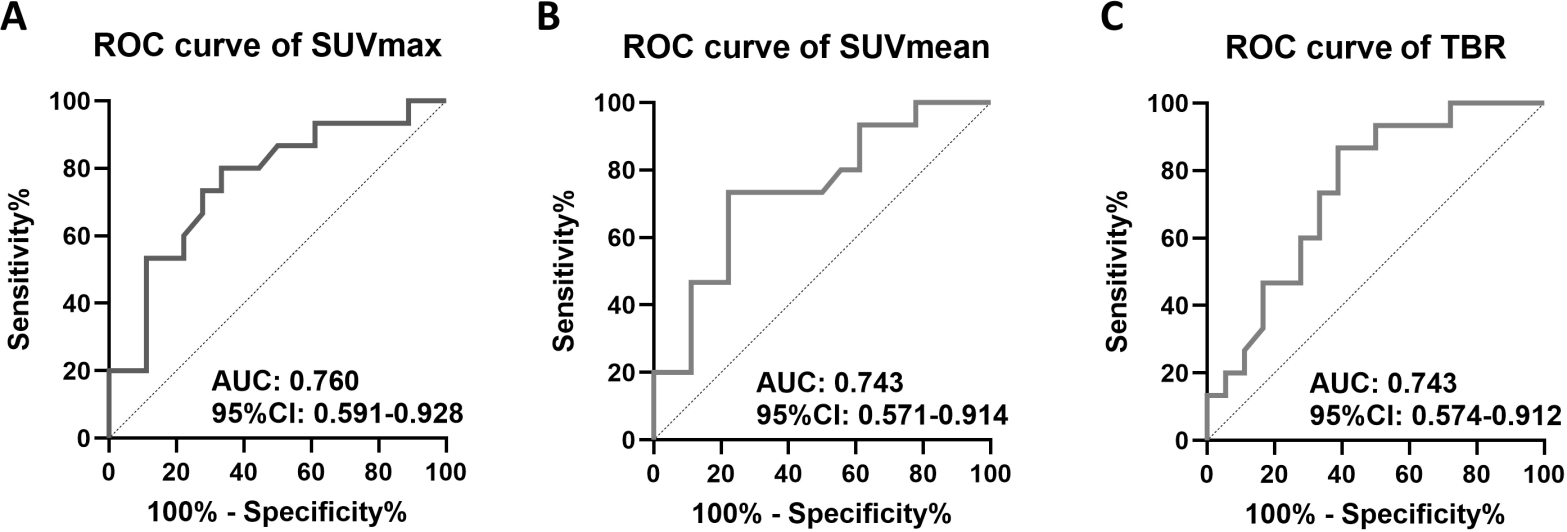
Receiver operator characteristic (ROC) analyses to distinguish active from inactive GCA by [^18^F]-FDG uptake of both optic nerves. **(A)** SUV_max_, **(B)** SUV_mean_, and **(C)** target-to-background ratio (TBR), which is calculated from SUV_max_/average SUV_mean_ of the vena cava. AUC, area under the curve; CI, confidence interval.

ROC analyses were calculated to determine [^18^F]-FDG uptake values of the ONs that can distinguish between active and inactive disease: the cutoff of the SUV_max_ was 10.9 with a sensitivity of 80.0% and a specificity of 66.7% [area under the curve (AUC) 0.760, 95% confidence interval (CI) 0.591–0.928, *p* = 0.0114]. The cutoff of the SUV_mean_ was 6.3 with a sensitivity of 73.3% and a specificity of 77.8% (AUC 0.743, 95% CI 0.571–0.914, *p* = 0.0179). The cutoff of the TBR was 7.5 with a sensitivity of 86.7% and a specificity of 61.1% (AUC 0.743, 95% CI 0.574–0.912, *p* = 0.0179) (**Figure 3**).

### Changes of [^18^F]-FDG uptake of the optic nerve in GCA do not depend on the type of immunosuppression

**Figure 4:**
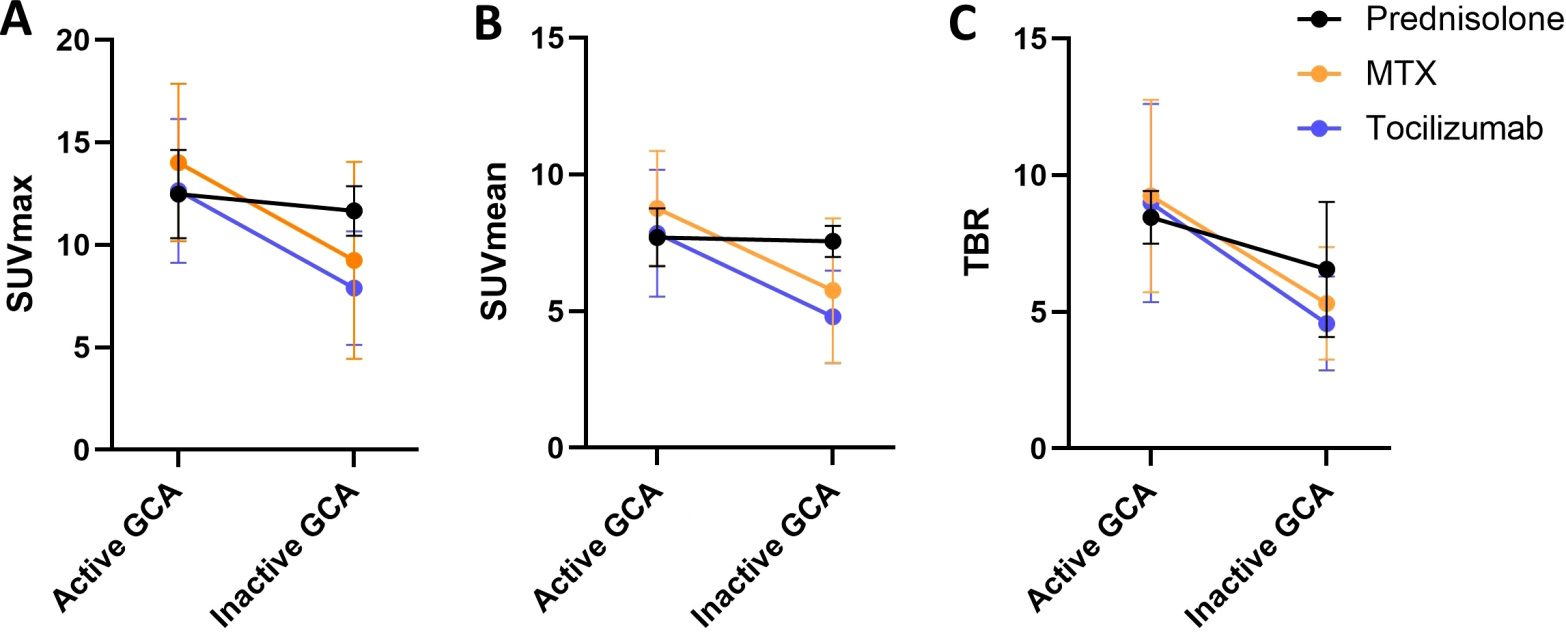
Change of [^18^F]-FDG uptake of both optic nerves depending on immunosuppressive treatment with prednisolone monotherapy (black; *n* = 2), methotrexate (MTX; orange; *n* = 4), or tocilizumab (purple; *n* = 9). **(A)** SUV_max_, **(B)** SUV_mean_, and **(C)** target-to-background ratio (TBR), which is calculated from SUV_max_/average SUV_mean_ of the vena cava. Shown are medians, and whiskers indicate interquartile ranges.

The patients were treated with either prednisolone monotherapy (*n* = 2), methotrexate (MTX; *n* = 4), or tocilizumab (*n* = 9). Patients who were not in remission at the time of the second [^18^F]-FDG-PET/CT were not included in this subgroup analysis. There were no differences between the change of the [^18^F]-FDG uptake of both ONs under the different treatments. Median ΔSUV_max_ (active minus inactive) values were as follows: −0.8 (IQR −1.5 to −0.2) in the prednisolone-treated group, −4.2 (−11.7–1.5) in the MTX-treated group, and −3.6 (−8.3 to −1.7) in the tocilizumab-treated group (**Figure 4A**). Median ΔSUV_mean_ values were as follows: −0.2 (−0.5–0.2) in the prednisolone-treated group, −2.3 (−6.6 to −0.2) in the MTX-treated group, and −2.4 (−5.5 to −1.1) in the tocilizumab-treated group (**Figure 4B**). Median ΔTBR values were as follows: −1.9 (−3.0 to −0.8) in the prednisolone-treated group, −2.7 (−8.6 to −0.5) in the MTX-treated group, and −2.8 (−7.2 to −1.7) in the tocilizumab-treated group (**Figure 4C**). No significant differences between the groups could be detected.

### [^18^F]-FDG uptake of the optic nerve in patients with GCA does not differ between the left and right side

**Figure 5:**
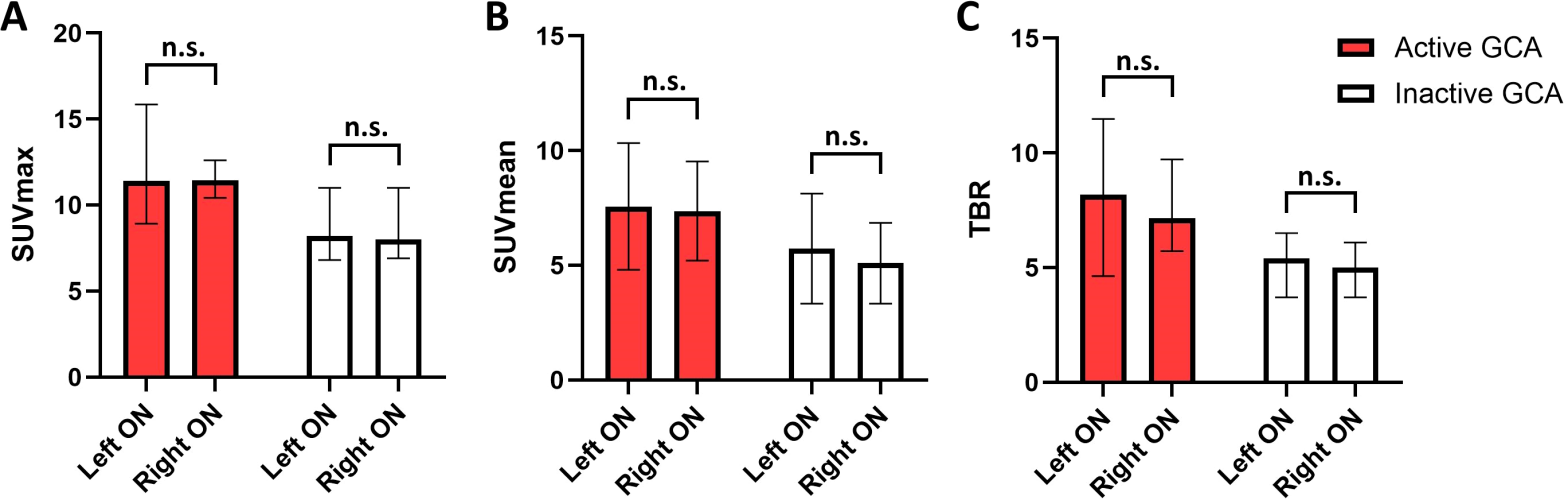
Comparison of [^18^F]-FDG uptake of the right versus the left optic nerve (ON) in patients with active giant cell arteritis (GCA; red bars; *n* = 18) and in inactive GCA (white bars; *n* = 15). **(A)** SUV_max_, **(B)** SUV_mean_, and **(C)** target-to-background ratio (TBR), which is calculated from SUV_max_/average SUV_mean_ of the vena cava. Shown are medians, and whiskers indicate interquartile ranges. *p*-values were calculated with Mann–Whitney *U* tests. n.s., not significant.

The [^18^F]-FDG uptake was measured in projection on the right ON and on the left ON separately. No significant differences of the SUV_max_, the SUV_mean_, or the TBR were detected between the right and left ON. Similar [^18^F]-FDG uptake on both sides of the ON was measured in patients with active and inactive GCA (**Figure 5**). Median SUV_max_ values in active GCA were as follows: 11.5 (IQR 10.4–12.6) on the right and 11.4 (8.9–15.9) on the left (*p* = 0.9938), median SUV_mean_ values in active GCA were as follows: 7.1 (6.3–8.2) on the right and 7.1 (5.4–10.0) on the left (*p* = 0.9938), and median TBR values in active GCA were as follows: 7.1 (5.7–9.7) on the right and 8.2 (4.6–11.5) on the left (*p* = 0.7965). Median SUV_max_ values in inactive GCA were as follows: 8.0 (6.9–11.0) on the right and 8.2 (6.8–11.0) on the left (*p* = 0.8459), median SUV_mean_ values in inactive GCA were as follows: 4.8 (4.0–7.0) on the right and 6.0 (4.2–7.2) on the left (*p* = 0.5596), and median TBR values in inactive GCA were as follows: 5.0 (3.7–6.1) on the right and 5.4 (3.7–6.5) on the left (*p* = 0.6602).

### [^18^F]-FDG uptake of the optic nerve in patients with GCA does not correlate with CRP-values

**Figure 6:**
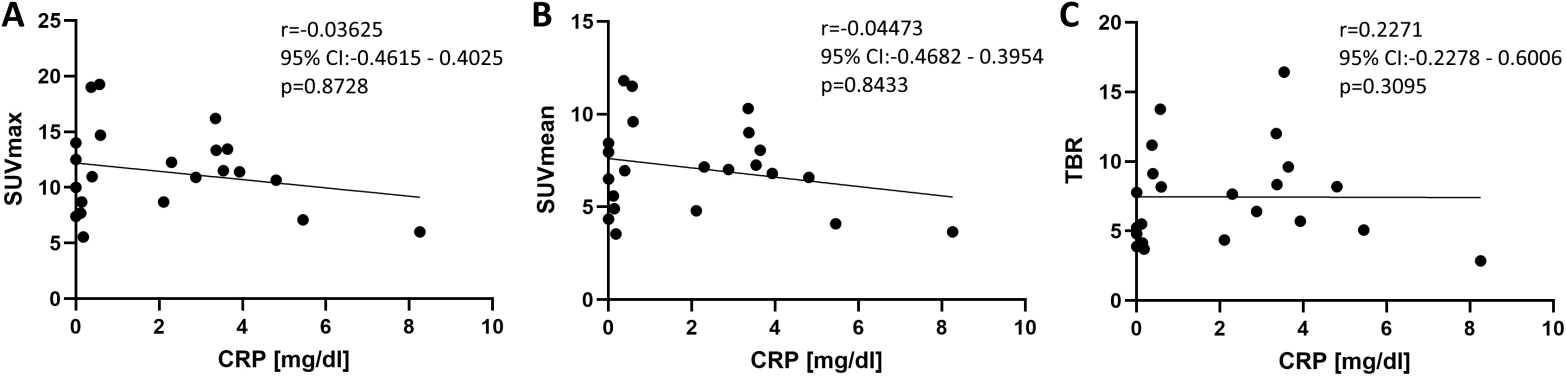
Correlation between [^18^F]-FDG uptake of both optic nerves. **(A)** SUV_max_, **(B)** SUV_mean_, and **(C)** target-to-background ratio (TBR). Calculations were done with a two-sided Spearman test.

No correlation could be detected between SUV_max_ of both ONs and the CRP value in peripheral blood from the same day (*r* = −0.0365, 95% CI −0.4615–0.4025, *p* = 0.8728), between SUV_mean_ and CRP (*r* = −0.04473, 95% CI −0.4682–0.3954, *p* = 0.8433), and between TBR and CRP (*r* = 0.2271, 95% CI −0.2278–0.6006, *p* = 0.3095) (**Figure 6**).

## Discussion

To our knowledge, this is the first study to describe inflammation of the ONs in GCA detected with [^18^F]-FDG-PET/CT. Although most of our patients had no visual symptoms, significantly higher [^18^F]-FDG uptake was observed, compared to a control group of individuals with untreated bronchial carcinoma. It seems likely that the ON inflammation is connected to GCA itself, as sufficient GCA treatment decreases the ON inflammation, although it remains higher than in the control group. This suggests a high prevalence of subclinical ON inflammation in GCA.

In the present study, the ON demonstrated markedly higher SUV_max_ values compared to all evaluated large-vessel territories. While vascular SUV_max_ values ranged approximately between 2 and 4, ON SUV_max_ exceeded 10, representing a substantial quantitative difference. Importantly, ON FDG uptake decreased following initiation of corticosteroid therapy, paralleling the reduction observed in inflamed vascular segments. It is well established that neural tissue exhibits physiological [^18^F]-FDG uptake due to its intrinsic glucose metabolism (12). Therefore, direct comparison between vascular wall uptake and neural tissue uptake must be interpreted cautiously. However, the magnitude of the observed difference and its dynamic reduction following corticosteroid therapy argue against purely physiological metabolic activity as the sole explanation. Physiological neural [^18^F]-FDG uptake would not be expected to demonstrate a rapid and consistent treatment response. Previous [^18^F]-FDG PET/CT studies in GCA have predominantly focused on large-vessel involvement (13–15), whereas data regarding orbital or ON metabolic alterations remain scarce. The present findings therefore suggest that ON metabolic changes may be underrecognized in routine PET assessment of GCA. If confirmed in larger cohorts, ON [^18^F]-FDG uptake may represent a potential imaging biomarker for the diagnosis of GCA and might contribute to risk stratification for visual complications.

Interestingly, no correlation between CRP levels and [^18^F]-FDG uptake of the ON was present in our analyses. Therefore, integrating [^18^F]-FDG-PET/CT assessment of the ON into diagnostic work-ups might be particularly helpful in identifying patients with active GCA without a significant increase in systemic inflammatory biomarkers. Of the patients with GCA, 5%–10% are reported to have normal inflammatory biomarkers in the blood despite active disease (16, 17).

Because of the limited spatial resolution of [^18^F]-FDG-PET/CT, it is not possible to determine which anatomical structure of the ON is exactly inflamed. However, orbital MRI in patients with GCA has revealed inflammation of the sheath of the ONs in 72% of cases, inflammation of branches of the ophthalmic artery in 44% of cases, and inflammation of the ON itself in 6% (18) of cases. Thus, [^18^F]-FDG uptake in the inflamed sheath may be the biological correlate of the corresponding imaging findings. This could explain the discrepancy between significant inflammation and the absence of ischemic optical symptoms in our cohort. In addition to the inflamed sheath, the high [^18^F]-FDG uptake in PET/CT observed in the ONs may reflect inflammation of perineural vessels, inflammatory extension to adjacent tissues, or metabolic alterations secondary to ischemic stress.

An MRI study described subclinical vessel wall inflammation of the ophthalmic artery in 45% of cases (19), supporting the assumption of a high rate of intraorbital subclinical inflammation in GCA. Additionally, subclinical inflammation of arteries can be detected without symptoms in other areas of the body, such as the intracranial region (20). The long-known high prevalence of subclinical vessel wall inflammation in patients with PMR (21) (then mainly named subclinical GCA) is consistent with the idea that numerous subclinical foci of inflammation are found in GCA.

As blood glucose levels can vary at each time point when a [^18^F]-FDG-PET/CT scan is performed, normalization of the SUV_max_ is necessary. Therefore, we calculated the TBR, which is SUV_max_ divided by the average SUV_mean_ of the vena cava, which makes the TBR values more reliable for the detection of hypermetabolism than the SUV_max_ or SUV_mean_.

Our control group consisted of patients with bronchial carcinoma who had not yet received (chemo) therapy. This group was selected because no vessel wall inflammation is expected (in contrast to patients with hematological neoplasms with potential spreading into the blood), leukocyte counts are unaltered due to their treatment-naïve status, and ON involvement is unlikely in this tumor entity. However, potential confounders for comparison like age differences, sex differences, systemic inflammatory status, and metabolic status cannot be excluded.

Limitations of our study include the small cohort size and its retrospective, single-center design. As only one patient experienced visual symptoms, the predictive value of ON [^18^F]-FDG uptake for ocular ischemic complications is limited. The data do not allow conclusions regarding the prognostic relevance of ON inflammation. Further studies should compare patients with GCA with visual symptoms (only one patient in the cohort suffered from an AAION) to patients without visual symptoms. Currently, no treatment or follow-up recommendations can be made based on the detection of subclinical ON inflammation. Additionally, a direct comparison of [^18^F]-FDG-PET/CT with MRI would be helpful to validate and correlate these findings.

In conclusion, ON inflammation, visualized by [^18^F]-FDG-PET/CT, appears to be more prevalent in GCA than previously assumed and may represent a useful tool to improve diagnostic accuracy and risk stratification in patients with GCA in the future.

## Data Availability

The raw data supporting the conclusions of this article will be made available by the authors, without undue reservation.

## Supplementary material

Supplementary Figure 1 Representative PET/CT scan to illustrate optical nerve measurements. Regions of interest (ROIs; red circles in the middle panel) were manually placed within the canalicular segment of the optic nerve, defined as the portion of the nerve located within the bony optic canal between the orbital apex and the intracranial exit. Care was taken to avoid spillover from adjacent orbital fat and intracranial structures.

Supplementary Figure 2 Median SUVmax values of different arteries in comparison to the optic nerves. Shown are medians, whiskers indicate inter quartile ranges. N = 18 for each region. * significant difference of the left or right optic nerve compared to each artery (segment).

## References

[1] González-Gay MÁ Heras-Recuero E García-Fernández A Blázquez-Sánchez T Torres-Roselló A Caraballo-Salazar C . Revisiting the epidemiology of giant cell arteritis. Clin Exp Rheumatol. (2025) 43:742–8. doi: 10.55563/clinexprheumatol/xc46ld, PMID: 40201967

[2] Muratore F Kermani TA Crowson CS Green AB Salvarani C Matteson EL . Large-vessel giant cell arteritis: a cohort study. Rheumatol (Oxford). (2015) 54:463–70. doi: 10.1093/rheumatology/keu329, PMID: 25193809 PMC4425829

[3] Cacoub P Vieira M Langford CA Tazi Mezalek Z Saadoun D . Large-vessel vasculitis. Lancet. (2025) 406:2017–32. doi: 10.1016/S0140-6736(25)01436-9, PMID: 40939604

[4] Solans-Laqué R de Escalante-Yanguela B Fonseca E Fraile G Martínez-Valle F Caminal L . Identification of risk factors for permanent visual loss in patients with giant cell arteritis. Eur J Intern Med. (2025) 139:106372. doi: 10.1016/j.ejim.2025.06.001, PMID: 40518343

[5] Liozon E Dalmay F Lalloue F Gondran G Bezanahary H Fauchais AL . Risk factors for permanent visual loss in biopsy-proven giant cell arteritis: A study of 339 patients. J Rheumatol. (2016) 43:1393–9. doi: 10.3899/jrheum.151135, PMID: 27134245

[6] Tucker SM Haas SJ Zaihra Rizvi T . Predictors of permanent vision loss in giant cell arteritis. Neuroophthalmology. (2024) 49:60–8. doi: 10.1080/01658107.2024.2389934, PMID: 40919082 PMC12409919

[7] Dejaco C Ramiro S Bond M Bosch P Ponte C Mackie SL . EULAR recommendations for the use of imaging in large vessel vasculitis in clinical practice: 2023 update. Ann Rheum Dis. (2024) 83:741–51. doi: 10.1136/ard-2023-224543, PMID: 37550004

[8] Guggenberger KV Pavlou A Cao Q Bhatt IJ Cui QN Bley TA . Orbital magnetic resonance imaging of giant cell arteritis with ocular manifestations: a systematic review and individual participant data meta-analysis. Eur Radiol. (2023) 33:7913–22. doi: 10.1007/s00330-023-09770-2=, PMID: 37256352 PMC11218900

[9] Petzinna SM Burg LC Terheyden JH Bauer CJ Karakostas P Behning C . Transorbital ultrasonography reveals persistent decrease in retinal artery flow and optic nerve diameter in treated giant cell arteritis. Rheumatol (Oxford). (2025), keaf456. doi: 10.1093/rheumatology/keaf456, PMID: 40880298

[10] Ponte C Grayson PC Robson JC Suppiah R Gribbons KB Judge A . 2022 American College of Rheumatology/EULAR classification criteria for giant cell arteritis. Ann Rheum Dis. (2022) 81:1647–53. doi: 10.1136/ard-2022-223480, PMID: 36351706

[11] Hellmich B Agueda A Monti S Buttgereit F de Boysson H Brouwer E . 2018 Update of the EULAR recommendations for the management of large vessel vasculitis. Ann Rheum Dis. (2020) 79:19–30. doi: 10.1136/annrheumdis-2019-215672, PMID: 31270110

[12] Jadvar H Subramaniam RM Berman CG Boada F Colletti PM Guimaraes AR . American college of radiology and society of nuclear medicine and molecular imaging joint credentialing statement for PET/MR imaging: brain. J Nucl Med. (2015) 56:642–5. doi: 10.2967/jnumed.115.155218, PMID: 25745088 PMC4465538

[13] Blockmans D de Ceuninck L Vanderschueren S Knockaert D Mortelmans L Bobbaers H . Repetitive 18F-fluorodeoxyglucose positron emission tomography in giant cell arteritis: a prospective study of 35 patients. Arthritis Rheumatol. (2006) 55:131–7. doi: 10.1002/art.21699, PMID: 16463425

[14] Nielsen BD Hansen IT Kramer S Haraldsen A Hjorthaug K Bogsrud TV . Simple dichotomous assessment of cranial artery inflammation by conventional 18F-FDG PET/CT shows high accuracy for the diagnosis of giant cell arteritis: a case-control study. Eur J Nucl Med Mol Imaging. (2019) 46:184–93. doi: 10.1007/s00259-018-4106-0, PMID: 30066157

[15] Galli E Muratore F Mancuso P Boiardi L Marvisi C Besutti G . The role of PET/CT in disease activity assessment in patients with large vessel vasculitis. Rheumatol (Oxford). (2022) 61:4809–16. doi: 10.1093/rheumatology/keac125, PMID: 35258570 PMC9707005

[16] Kyle V Cawston TE Hazleman BL . Erythrocyte sedimentation rate and C reactive protein in the assessment of polymyalgia rheumatica/giant cell arteritis on presentation and during follow up. Ann Rheum Dis. (1989) 48:667–71. doi: 10.1136/ard.48.8.667, PMID: 2782977 PMC1003844

[17] Salvarani C Hunder GG . Giant cell arteritis with low erythrocyte sedimentation rate: frequency of occurence in a population-based study. Arthritis Rheumatol. (2001) 45:140–5. doi: 10.1002/1529-0131(200104)45:2<140::aid-anr166>3.0.co;2-2 11324777

[18] Guggenberger KV Vogt ML Song JW Weng AM Fröhlich M Schmalzing M . Intraorbital findings in giant cell arteritis on black blood MRI. Eur Radiol. (2023) 33:2529–35. doi: 10.1007/s00330-022-09256-7, PMID: 36394601 PMC10017783

[19] Geiger J Ness T Uhl M Lagrèze WA Vaith P Langer M . Involvement of the ophthalmic artery in giant cell arteritis visualized by 3T MRI. Rheumatol (Oxford). (2009) 48:537–41. doi: 10.1093/rheumatology/kep011, PMID: 19233887

[20] Donaldson L Nanji K Rebello R Khalidi NA Rodriguez AR . Involvement of the intracranial circulation in giant cell arteritis. Can J Ophthalmol. (2020) 55:391–400. doi: 10.1016/j.jcjo.2020.04.002, PMID: 32416931

[21] Salvarani C Padoan R Iorio L Tomelleri A Terrier B Muratore F . Subclinical giant cell arteritis in polymyalgia rheumatica: Concurrent conditions or a common spectrum of inflammatory diseases? Autoimmun Rev. (2024) 23:103415. doi: 10.1016/j.autrev.2023.103415, PMID: 37625672

