# Supplementary figures and images for: [18F]-FDG-PET/CT detects subclinical optic nerve inflammation in giant cell arteritis
Source: Front Immunol. 2026 Mar 18;17:1802935. doi: 10.3389/fimmu.2026.1802935 (PMC13038984; doi:10.3389/fimmu.2026.1802935)

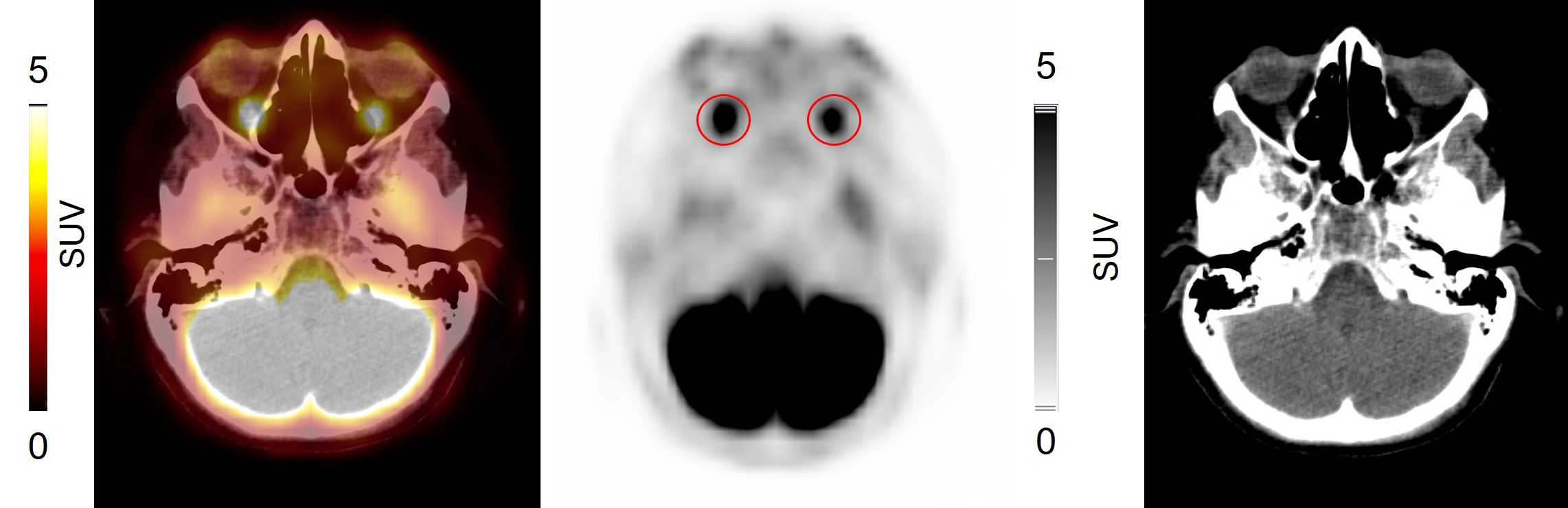

Supplement: Supplementary Figure 1 — Representative PET/CT scan to illustrate optical nerve measurements. Regions of interest (ROIs; red circles in the middle panel) were manually placed within the canalicular segment of the optic nerve, defined as the portion of the nerve located within the bony optic canal between the orbital apex and the intracranial exit. Care was taken to avoid spillover from adjacent orbital fat and intracranial structures. [file Image1.jpeg]

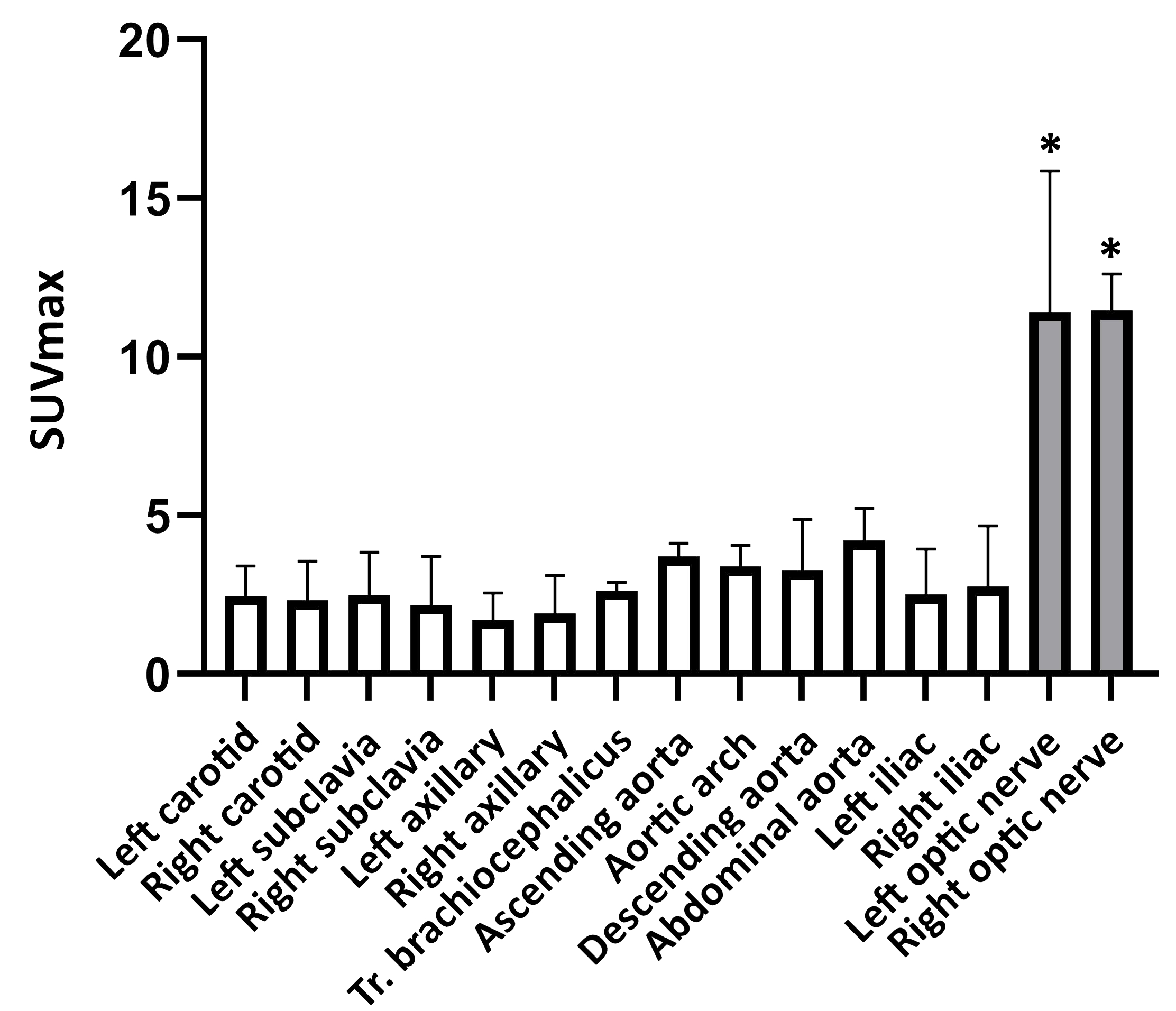

Supplement: Supplementary Figure 2 — Median SUVmax values of different arteries in comparison to the optic nerves. Shown are medians, whiskers indicate inter quartile ranges. N = 18 for each region. * significant difference of the left or right optic nerve compared to each artery (segment). [file Image2.jpeg]
